# Case report: Highly differentiated endometrial adenocarcinoma that collided with uterine cervical carcinosarcoma

**DOI:** 10.3389/fmed.2023.1044196

**Published:** 2023-01-26

**Authors:** Xue Fan, Haoyue Hu, Yang Liu, Songtao Tan, Meng Xie, Fang Zhang

**Affiliations:** ^1^Institute of Lung Cancer, Guangdong Provincial People’s Hospital (Guangdong Academy of Medical Sciences), Southern Medical University, Guangzhou, China; ^2^Sichuan Cancer Center, Sichuan Cancer Hospital and Institute, Medicine School of University of Electronic Science and Technology, Chengdu, China; ^3^Department of Medical Oncology, Sichuan Cancer Center, Sichuan Cancer Hospital and Institute, Medicine School of University of Electronic Science and Technology, Chengdu, China; ^4^Department of Pathology, Sichuan Cancer Center, Sichuan Cancer Hospital and Institute, Medicine School of University of Electronic Science and Technology, Chengdu, China; ^5^West China School of Medicine, Sichuan University, Chengdu, China; ^6^Department of Pharmacy, Southwest Medical University, Luzhou, China

**Keywords:** collision tumor, endometrial adenocarcinoma, therapy, cervical carcinosarcoma, pathology

## Abstract

Uterine collision tumor is a rare pathological type composed of two or more malignant tumors. The components of these malignant tumors do not have histological mixing and are separated by the normal mesenchyme. Collision cancer is very rare, with uterine collision tumors being even rarer. Only a few cases have been reported in relation to uterine collision tumors. At present, there is no standard treatment guideline for uterine collision tumors, and a comprehensive treatment composed of surgery, radiotherapy, and chemotherapy is suggested. In this study, we report a 54-year-old female patient diagnosed with highly differentiated endometrial adenocarcinoma with cervical carcinosarcoma. The endometrial adenocarcinoma component invaded the deep myometrium (> 1/2 layer), involving the cervical glands and interstitium. The regional lymph node metastasis from endometrial adenocarcinoma was also detected. The patient underwent “transabdominal tumor cytoreduction (total hysterectomy + right adnexal resection + greater omentectomy + pelvic lymph node dissection + para-aortic lymph node dissection) + pelvic adhesion release.” In addition, she has completed adjuvant radiotherapy and chemotherapy. After reviewing previous reports of collision tumors in different positions of the uterus, we found that collision tumors between the cervix and uterine body are very uncommon. In addition, we have not found any reports on the metastasis of sarcoid components, no matter what the composition is.

## Introduction

Endometrial adenocarcinoma is a gynecological malignant tumor with a very high incidence that usually occurs in postmenopausal women (1). In 2018, there were more than 380,000 new cases of endometrial carcinoma worldwide, with endometrial adenocarcinoma accounting for about 85% of cases (2, 3). Endometrial adenocarcinoma is mainly caused by the abnormal proliferation of endometrial epithelial cells. It can be divided into highly differentiated adenocarcinoma, moderately differentiated adenocarcinoma, and poorly differentiated adenocarcinoma. The highly differentiated adenocarcinoma is a special type of pathology that is less malignant and relatively easy to treat. Carcinosarcoma is an uncommon malignancy in women that occurs in the uterine cervix. Cervical sarcomas accounted for 0.005% of all cervical malignancies. Cervical carcinosarcoma is one of its types, and the literature on cervical carcinosarcoma is currently limited to case reports (4). It is also named a malignant mixed mesodermal tumor, which is composed of a carcinomatous component and a sarcomatous component; malignant mixed Müllerian tumor; or metaplastic carcinoma (5). In this study, we report a 54-year-old female who was diagnosed with highly differentiated endometrial adenocarcinoma with cervical carcinosarcoma.

## Case report

**FIGURE 1:**
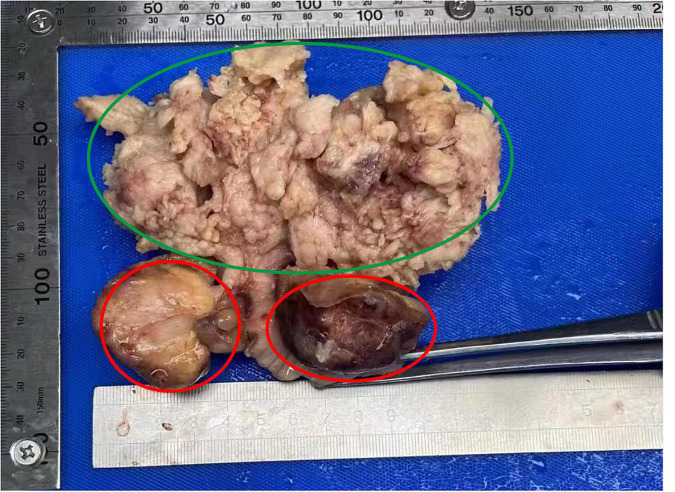
Macroscopic appearance of the specimen indicating endometrial carcinoma of uterine corpus (green circle) and cervical carcinosarcoma (red circle).

**FIGURE 2:**
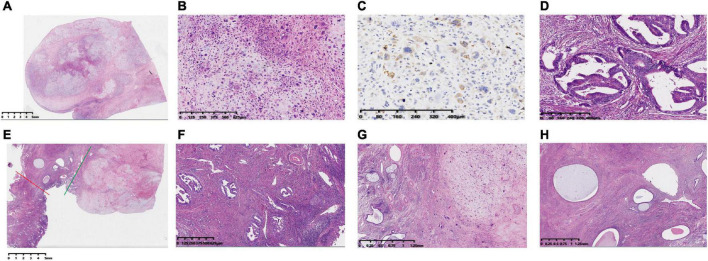
Histopathology of the hysterectomy specimen HE stained and immunohistochemical staining of EMA in cervical carcinosarcoma. **(A)** Panoramic view of the histological appearance of a cervical cancer sarcoma under low magnification (HE). **(B)** The components of carcinoma and sarcoma are closely related and are mixed together under high-magnification microscopy. The morphology of cervical cancer is mainly sarcomatous with chondrogenic differentiation. **(C)** Immunohistochemistry of a cervical mass showing that the tumor cell cytosol was EMA positive. **(D)** A uterine mass showing endometrial adenocarcinoma. **(E)** The red line to the left indicates the uterine mass and the green line to the right indicates the cervical mass, both of them meeting at the endocervix, with normal endocervical glands and interstitium visible at low magnification (between the red and green lines). **(F)** High-magnification of a cervical mass involving the cervical mesenchyme with surface glandular phosphorylation—magnification. **(G)** Low-magnification microscopy of a cervical mass involving the endocervix—magnification. **(H)** Low-magnification view of the intersection of endometrial adenocarcinoma and cervical carcinosarcoma in the body of the uterus. The collision of these two tumors shows a clear border.

A 54-year-old female patient presented with “irregular vaginal bleeding” in June 2021 without specific treatment. In November 2021, the patient visited our hospital with worsening vaginal bleeding symptoms for further treatment. She had menarche at the age of 14 years and menopause 6 years ago with previous cycles of 5–6 days/28 days with normal flow. No significant past or family history was reported. Vaginal examination showed a polypoid mass with a diameter of about 5 cm in the cervix and free parametrial tissue on both sides. Abdominal MRI showed that the cervix was obviously thickened, mainly on the posterior wall, with the thickest part being about 7.2 cm, and the lesion involved the upper vaginal segment downward. The myometrium is also obviously thickened. A vaginal tissue biopsy showed scattered heterogeneous cells, and combined with HE morphology and immunophenotype, mesenchymal-derived sarcoma was considered. The biopsy tissue tumor sample was small, so it was suggested to send further examination after complete excision for clarification. Immunohistochemistry of tumor cells was positive for EMA, P63, vimentin, desmin, SMA, Ki-67, ER, and caldesmon and negative for Ckpan (AE1/AE3), P40, actin, S100, MyoD1, myoglobin, and PR. In November 2021, she underwent “transabdominal tumor cytoreduction (total hysterectomy + right adnexal resection + greater omentectomy + pelvic lymph node [LN] dissection + para-aortic LN dissection) + pelvic adhesion release.” When the uterus is cut open, a cervical tumor (5 cm × 4.5 cm × 4 cm) and uterine tumor (6.8 cm × 6 cm × 4 cm) were seen (Figure 1). Routine examination revealed the following diagnoses: (1) left internal iliac and foramen occulans LN 1/6, right deep femoral LN 1/10, and para-aortic LN 1/11, showing the characteristic of tumor metastasis from endometrial adenocarcinoma; (2) left external iliac LN 1, left total iliac LN 6, left deep femoral LN 5, right external iliac LN 1, right total iliac LN 3, and right internal iliac and foramen occulans LN 1, all showing reactive hyperplasia; and (3) large omentum, but did not show any cancer involvement. Postoperative pathological diagnoses are as follows: (1) the “body of the uterus” is a highly differentiated endometrioid adenocarcinoma (Figures 2D, F), infiltrating deep myometrium (> 1/2 layer), involving the cervical glands and interstitium, without definite nerve invasion or choroidal carcinoma embolism; immunohistochemistry is recommended for follow-up treatment. No definite tumor residue was seen in the vaginal section or the left and right parametrial sections. (2) No tumor involvement was seen in the right adnexa. The first supplementary report revealed a cervical tumor, combined with HE morphology and immunophenotype, and the lesion was consistent with carcinosarcoma (Figure 2B), a sarcomatous component with chondrogenic differentiation. Immunohistochemistry of tumor cells was positive for EMA (Figure 2C), S100, caldesmon, calponin, P16, MDM2, CDK4, CD99, ER, Brg-1 (SMARCA4), Ki67, CD10, and Fli-1 and negative for Ckpan (AE1/AE3), CK8/18, CK7, desmin, PAX-8, SMA, P40, actin, PR, NKX2.2, WT1, MyoD1, myogenin, SATB2, and ERG. The cervical and uterine body lesions were observed microscopically as two different tumor sites. Microscopically, endometrial adenocarcinoma and cervical carcinosarcoma collide at the cervix and show clear boundaries (Figures 2A, E, G, H). The FIGO stage was IIIc. Cycle 1 chemotherapy (paclitaxel 250 mg ivgtt d1 + carboplatin 500 mg ivgtt d1) was administered on 17 December 2021 and later administered doxorubicin 100 mg ivgtt d1 + carboplatin 300 mg ivgtt d1 in cycle 2 due to the patient’s heavy treatment response. On 21 February 2022, the patient completed 28 external pelvic irradiation radiotherapy and 2 intracavitary backloading treatments. The patient continued with 3–6 cycles of chemotherapy (doxorubicin 100 mg ivgtt d1 + carboplatin 300 mg ivgtt d1). She remained healthy with no evidence of recurrence for 13 months after surgery.

## Discussion

Collision tumors refer to the simultaneous occurrence of two or more malignant tumors of different tissue origins in the same organ or viscera. Tumors collide with each other; however, there is no obvious migration transition between the two. Collision tumors have been described in organs such as the thyroid, lung, stomach, and, rarely, in the uterus. As mentioned previously, collision tumors are difficult to diagnose by imaging examination, and most cases can only be diagnosed by postoperative pathology. To the best of our knowledge, this is the first case to describe a highly differentiated endometrial adenocarcinoma that collided with uterine cervical carcinosarcoma.

The solid components of uterine collision tumors are composed of two or more tumor components. According to different collision combinations, a variety of component types can appear. The etiology of uterine collision tumors is unclear; however, its etiology may be related to the etiology of the included tumors. According to the anatomical location of the uterus, uterine tumors are mostly divided into cervical tumors (cervical cancer and cervical fibroids) and uterine corpus tumors (endometrial cancer, uterine fibroids, and uterine sarcomas). Collision tumors of the uterus are rare, with only a few such cases reported thus far. We have reviewed six collision tumors in different locations of the uterus (Table 1). Ki-Seok Jang reported that a 70-year-old patient with a collision tumor of the uterine corpus consisted of a malignant mixed Müllerian tumor, papillary serous carcinoma, and endometrioid adenocarcinoma (6). Yoshihisa Takahashi reported that a 68-year-old woman underwent a hysterectomy and bilateral salpingo-oophorectomy and found the pathological diagnosis was collision tumor, including hepatoid carcinoma and carcinosarcoma (7). Shobhna Sharma reported a collision tumor of endometrial stromal sarcoma and endometrioid adenocarcinoma in a 65-year-old female (8). All the above three cases contain collision components of carcinoma and sarcoma, all of which are located in the uterine body. Anne-Marie reported two cases of collision tumors occurring only in the cervix. One case is a 52-year-old woman who underwent a radical hysterectomy and lymphadenectomy. Histological sections from different regions of the tumor showed adenocarcinoma and squamous cell carcinoma. Another case is a 62-year-old woman who underwent radical hysterectomy and lymphadenectomy and showed adenocarcinoma and squamous cell carcinoma (9). Nadeem Tanveer reported a rare collision tumor of cervical squamous cell carcinoma and endometrial stromal sarcoma (10). The location of this case and the collision cancer reported by us are both in the cervix and uterus, and more importantly, the patient we reported is a rare carcinosarcoma of the cervix. The collision tumors we have reviewed had both carcinomatous and sarcoid components. The regional LN metastases were found in 4 cases, but these metastatic LNs had only a carcinomatous component. The aggressive and metastatic components of collision tumors were thought to depend on their original biological behavior. But this seems to be contradictory to the fact that sarcoma is more likely to invade and metastasize. Therefore, we should find whether the stage of sarcoma is earlier than that of cancer. As there are too few case reports of collision cancer, we need more data for further studies for a more in-depth discussion.

**TABLE 1:** Summary of previously reported collision tumors of the uterine.

Location	Carcinoma	Sarcoma	Regional lymph node	Ref.
Uterine corpus	Hepatoid carcinoma	Carcinosarcoma	Non	(7)
	Papillary serous carcinoma, endometrioid adenocarcinoma	Malignant mixed müllerian tumor	Papillary serous carcinoma	(6)
	Endometrioid adenocarcinoma	Endometrial stromal sarcoma	Non	(8)
Cervix	Adenocarcinoma, squamous cell carcinoma		Squamous cell carcinoma	(9)
	Adenocarcinoma, squamous cell carcinoma		Adenocarcinoma	(9)
Uterine corpus and cervix	Squamous cell carcinoma of cervix	Endometrial tromal sarcoma (high grade) uterus	Non	(10)
	Endometrial adenocarcinoma	Carcinosarcoma of cervix	Endometrial adenocarcinoma	Present case

At present, there is a lack of specific clinical manifestations and imaging examinations for uterine collision tumors. Most patients with uterine collision tumors can be diagnosed by histopathological examination after surgery. The mechanism of collision tumors is unclear and controversial. The appearance of the collision tumor was an accidental event, and two morphologically distinct tumors may develop adjacently due to the same oncogenic factors in the region, such as radiation, malignant transformation, and changes in the microenvironment (11). Masuyama reported a woman diagnosed with a collision tumor composed of three histologically distinct neoplasms in the uterine corpus, namely, endometrioid carcinoma, undifferentiated carcinoma, and choriocarcinoma. The patient underwent a hysterectomy, bilateral adnexectomy, and pelvic LN dissection, followed by adjuvant chemotherapy. The tumor markers were within the normal limits, and no relapses of the cancer were observed during 1-year follow-up (12). Our patient also underwent six cycles of adjuvant chemotherapy, but the prognosis requires a longer follow-up. Previous cases have shown that each component of a colliding tumor occurs coincidentally, with no connection, and that biological behavior depends on its own tumor characteristics. The clinical outcome of endometrial adenocarcinoma is generally favorable because most women have an early-stage disease that can be treated with radical surgery and/or radiation therapy (RT). However, for those with relapsed or advanced disease, the prognosis is less favorable. In this case, treatment may include hormone therapy or chemotherapy, sometimes in combination with further retroviral therapy and possibly surgery. The clinical presentation of carcinosarcoma is usually more aggressive, often presenting as an advanced disease. Systemic chemotherapy is usually required after cytoreductive surgery. There are no clear treatment guidelines for uterine collision tumors. In clinical practice, the treatment principle of this disease is similar to that of a single uterine tumor. Comprehensive treatment is adopted mainly by surgery and radiotherapy or supplemented by chemotherapy.

However, doctors should choose specific treatment strategies according to the location, size, and stage of the tumor. The diagnosis of a collision tumor basically depends on the postoperative pathological decision because it requires broad gross sampling. In this case, the preoperative cervical mass biopsy showed cervical sarcoma, and the imaging examination showed that the myometrium was thickened. The preoperative diagnosis of collision tumors is difficult when these tumor components are closely located, and doctors may confuse the diagnosis, leading to an erroneous diagnosis. For correct diagnosis, doctors need to be differentiated between mixed tumors, compound tumors, and multiple primary cancers. Careful pathologic examination allows an accurate tumor stage for each tumor and histologic type, which has important implications for patient management and prognosis.

In general, uterine collision tumors are a rare disease with few reports in the literature. Therefore, clinicians, pathologists, and imaging physicians should improve their understanding of the disease, accurately diagnose the disease, and formulate a standardized diagnosis and treatment plan. The mechanism of uterine collision tumors also needs to be further explored. It is necessary to strengthen molecular biology research and multidisciplinary cooperation to provide new ideas for the treatment of uterine collision tumors.

## Data availability statement

The original contributions presented in this study are included in this article/supplementary material, further inquiries can be directed to the corresponding author.

## Ethics statement

Written informed consent was obtained from the individual(s) for the publication of any potentially identifiable images or data included in this article.

## Author contributions

XF: conceptualization, methodology, and writing—original draft. FZ: data curation and review and editing. HH: investigation and writing—original draft. YL: resources and supervision. ST: validation. MX: revision of the manuscript. All authors contributed to the article and approved the submitted version.
